# Ten years of online incident reporting and learning using CPiRLS: implications for improved patient safety

**DOI:** 10.1186/s12998-023-00477-1

**Published:** 2023-02-15

**Authors:** Mark Thomas, Gabrielle Swait, Rob Finch

**Affiliations:** 1 Institute of Health and Social Care, London South Bank University, London, UK; 2 Royal College of Chiropractors, Chiltern House, 45 Station Road, Henley on Thames, Oxfordshire, RG9 1AT UK

**Keywords:** Chiropractic, Manual therapy, Incident reporting, Patient safety, Risks, Safety incident, Adverse events

## Abstract

**Background:**

Safety incident (SI) reporting and learning via incident reporting systems (IRSs) is used to identify areas for patient safety improvement. The chiropractic patient incident reporting and learning system (CPiRLS) is an online IRS that was launched in the UK in 2009 and, from time to time, has been licensed for use by the national members of the European Chiropractors' Union (ECU), members of Chiropractic Australia and a Canada-based research group. The primary aim of this project was to analyse the SIs submitted to CPiRLS over a 10-year period to identify key areas for patient safety improvement.

**Method:**

All SIs reported to CPiRLS between April 2009 and March 2019 were extracted and analysed. Descriptive statistics were used to describe: (1) the frequency of SI reporting and learning by the chiropractic profession, and (2) the character of reported SIs. Key areas for patient safety improvement were developed following a mixed methods approach.

**Results:**

A total of 268 SIs were recorded on the database over the 10-year period, 85% of which originated from the UK. Evidence of learning was documented in 143 (53.4%) SIs. The largest subcategory of SIs related to post-treatment distress or pain (n = 71, 26.5%). Seven key areas for patient improvement were developed including: (1) patient trip/fall, (2) post treatment distress/pain, (3) negative effects during treatment, (4) significant post-treatment effects, (5) syncope, (6) failure to recognize serious pathology, and (7) continuity of care.

**Conclusion:**

The low number of SIs reported over a 10-year period suggests significant under-reporting, however, an upward trend was identified over the 10-year period. Several key areas for patient safety improvement have been identified for dissemination to the chiropractic profession. Improved reporting practice needs to be facilitated to improve the value and validity of reporting data. CPiRLS is important in identifying key areas for patient safety improvement.

**Supplementary Information:**

The online version contains supplementary material available at 10.1186/s12998-023-00477-1.

## Background

Improvement of patient safety has long been a priority [1, 2]. One means of addressing this is the reporting of, and learning from, safety incidents that have arisen. A safety incident (SI) is any type of deviation from normal clinical care that may occur and that has the potential to cause patient harm [3]. SIs are broad-ranging and include, for example, errors or delays in diagnosis or referral, patient accidents while in the clinic setting and documentation errors, as well as adverse events (AEs). Within the manual therapy context, the term AE has a narrower definition and refers to negative outcomes associated with treatment, although the relationship may not be causative [4].

Incident reporting systems (IRSs), are used to record and review SIs to facilitate improvement in patient safety and can vary in their aim, design and scale [3]. IRSs have been shown to identify safety needs, enabling improved clinical settings and processes [5], however, improved patient safety outcomes have not yet been established [6, 7]. Despite limited evidence of the effectiveness of IRSs, healthcare organisations have been utilising them to direct priorities for patient safety [7]. A recent analysis of 10 years of accumulated serious SIs reported in medical emergency units enabled the identification of key areas of risk and actions that may be taken to mitigate these and prevent future SI occurrence [5].

The amount of learning drawn from IRSs is variable, with the focus too often being on reporting [8]. The concept of learning from IRSs is ill-defined and there is a lack of literature on learning from such systems. The common practice of reviewing SIs and issuing patient safety reports may not be enough as learning is a complex, social process [8]. In England, a new national syllabus for enhancing patient safety in healthcare includes learning from patient safety incidents as a key domain [9], emphasising the importance of using reported SIs.

The majority of chiropractors do not work in large organisations with established clinical governance structures in place [10]. Furthermore, there is no systemic oversight of safety performance and limited opportunities for the profession to identify or learn from SIs. Thus, engagement with IRSs has a role to play in the development of a robust safety and governance culture within the chiropractic profession.

While benign AEs e.g. soreness or dizziness following manual therapy are a common occurrence, observed in approximately 30–50% of treatments for spinal pain [11], the risks of significant harm associated with manual therapy are low [12, 13]. Serious AEs (i.e. those resulting in serious injury or death) are extremely rare and a causal relationship with manual therapy has not been established [14, 15]. The detection of serious AEs associated with manual therapy is a limitation of most research designs due to their rarity. However, IRSs can play a role in their investigation [4], potentially identifying serious incidents and any contributing factors, and enabling learning and subsequent behaviour or system change to occur.

To date, within manual therapy, patient safety often appears to be focused on AEs and there is little understanding about the other types of SIs that can occur in chiropractic practice. Only case reports exist within the manual therapy literature in relation to SIs that do not result in harm but have the potential to do so i.e. ‘near misses’ [16, 17]. IRSs focused on learning can encourage the reporting of near misses as well as the identification of SIs that have the potential to occur in the future.

The chiropractic patient incident reporting and learning system (CPiRLS) is an IRS that enables chiropractors to report and learn from SIs [18]. Chiropractors are encouraged to report any SIs resulting in harm or potential harm, prompting reflection and/or discussion with others. CPiRLS was developed and launched in the UK by the Royal College of Chiropractors (RCC) in 2009 [18] and is currently accessible to all chiropractors in the United Kingdom (UK). It is regularly promoted to UK-based chiropractors in RCC literature and at RCC events. During the period under report, CPiRLS was licensed for use to the national members of the European Chiropractors’ Union (from 2012), to members of Chiropractic Australia (from 2018) and to a Canada-based research group (2015/16; [19]). These organisations were responsible for promoting its use in their jurisdictions. CPiRLS is a secure and anonymous online system designed to replace and unify two previous UK paper-based versions [18, 20].

Following the launch of the CPiRLS, internal SI monitoring and analysis resulted in the issue of two safety notices (‘Safer Practice Notices’) detailing the risks of rib fracture and falls respectively [21]. As the number of reported SIs grows, CPiRLS provides an increasingly valuable dataset for analysis of SI reporting by the chiropractic profession. The aim of this study was to analyse the SIs submitted to CPiRLS over a 10-year period (2009 to 2019) in order to enhance patient safety. The objectives were to: (1) Review the frequency of SI reporting and learning by the chiropractic profession, (2) Characterise the nature of the reported SIs, and (3) Identify key areas for patient safety improvement.

## Methods

### CPiRLS database

CPiRLS (accessible at https://cpirls.org/) is owned and maintained by the RCC. Access to CPiRLS content is password protected. CPiRLS automatically records the country of origin of each report. SIs are reported anonymously by chiropractors, who cannot be identified within the system.

SIs are initially recorded as red (actually occurred), amber (near miss) or green (potential to occur). Data captured for each SI includes: (1) Patient demographics i.e. patient age and gender, (2) SI classification and description, (3) Actions following the SI, (4) Details of (potential) patient harm, and (5) possible contributing factors. A combination of selection from drop-down categories and free text entry is used. An example of the data entry for the reporting of a red incident is demonstrated in Additional file 1.

### Analysis

The CPiRLS database was accessed, and data extracted into Microsoft Excel relating to all SIs submitted between April 2009 and March 2019. Frequency statistics were used to characterise the data captured for the frequency and nature of reported SIs. Documented indicators of learning from SIs were identified by one researcher (MT) by reviewing the “Describe the actions taken immediately and in the longer term” free text section.

A cross-sectional, mixed methods approach was used to identify key areas for patient safety improvement. Initially the most frequent subcategories were identified and reviewed with the additional use of keyword searches within the database e.g. for patient trip or fall the keywords ‘trip’ or ‘fall’ or ‘stumble’ were searched.

**Fig. 1:**
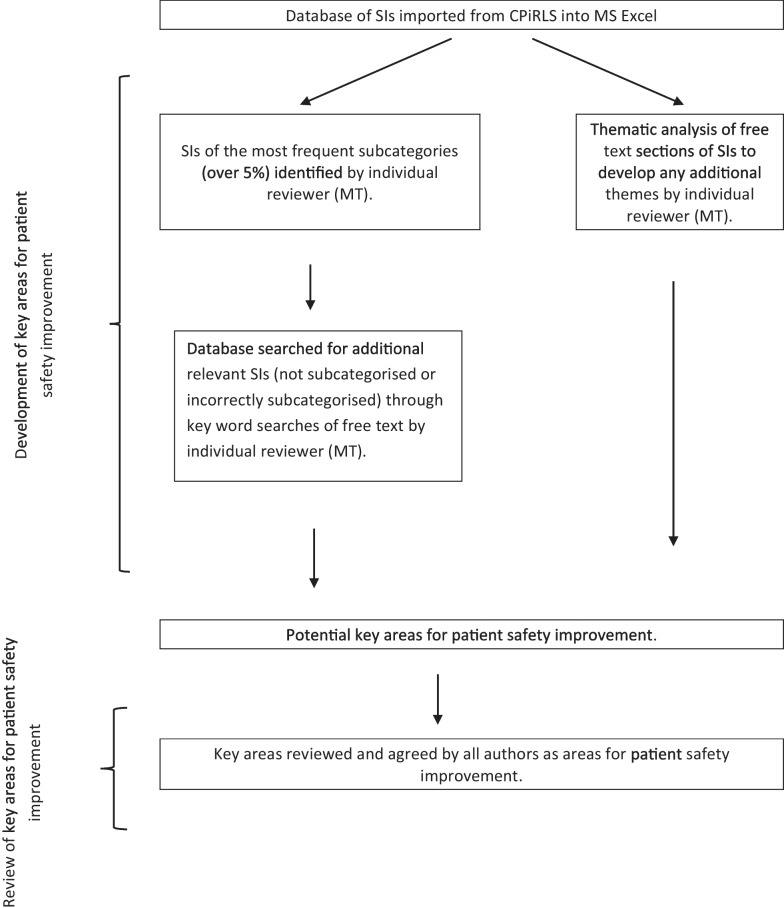
Methodology for data processing, analysis and identification of key areas for patient safety improvement

In addition, a thematic analysis was carried out to account for any key areas of patient safety improvement not currently defined as a subcategory on the CPiRLS database. The free-text boxes of “What happened - give details, including people and/or equipment involved” and “Why and how it happened - describe the sequence of events and possible causes” were analysed to develop themes on patient safety improvement by one researcher (MT). The coding was verified by another researcher (GS) and the themes were agreed by all authors. The key areas for patient safety improvement were then reviewed and agreed by all the authors and accepted if they comprised of: (1) a significant number of SIs represented (over 5%), and (2) sufficiently rich data to make recommendations with the potential to result in patient safety improvement. The data processing and analysis method is illustrated with a flow chart in Fig. 1.

## Results

### Frequency of incident reporting and learning by the chiropractic profession

A total of 268 SIs were reported over the ten year period, with an average 30.5% increase over whole years (2010–2018), demonstrating an upward trend over time (Additional file 2). Table 1 provides the distribution of country of origin of the SIs reported, indicating that the majority (85%) were from the UK. The SIs involved 148 (58.2%) female and 86 (32.1%) male patients and a further 34 (12.7%) with no gender specified. SIs were recorded representing patients in all age groups (Additional file 2) with a modal patient age group of 55–64 years old with 54 (20.1%) SIs recorded. Six (2.2%) SIs were recorded for patients under the age of 16.

**Table 1:** Country of origin of reported incidents

Country	Number of reported incidents
Australia	2 (0.7%)
Belgium	19 (7.1%)
Netherlands	2 (0.7%)
Norway	1 (0.4%)
Canada	3 (1.1%)
Sweden	13 (4.9%)
UK	228 (85.1%)
Total	268

A clear and documented indicator of learning from the reporting chiropractor was present in 143 (53.4%) SIs. Documented indicators of learning included personal reflection, discussion of change of practice or policy, additional training, discussion with others as well as completion of a clinical audit cycle.

### Characterisation of safety incidents reported

Table 2 provides the categorisation and numbers of SIs reported, of note one fifth of SIs were not assigned to a category or subcategory. ‘Treatment/Management’ was the largest category, accounting for over half of all SIs. The largest subcategory involved patients experiencing post-treatment distress/pain.

**Table 2:** Frequency of safety incident categories

SI category	SI subcategory	Number of SIs
Accidents/Equipment/Infrastructure	Patient trip/fall	23 (8.6%)
	Equipment malfunction	7 (2.7%)
	Failure to use equipment appropriately	7 (2.7%)
	Health and Safety measures inadequate	5 (1.9%)
	Failure to dispose of sharps and clinical waste appropriately	1 (0.4%)
	Exposure to blood	2 (0.7%)
	Exposure to harmful substances	1 (0.4%)
	Other	3 (1.1%)
	Unspecified	1 (0.4%)
	Subtotal	50 (18.6%)
Documentation	Patient record inadequate	3 (1.1%)
	Failure to document diagnosis/prognosis	1 (0.4%)
	Patient record misplaced	3 (1.1%)
	Records confused, treated wrong patient	5 (1.9%)
	Failure to gain consent	1 (0.4%)
	Other	5 (1.9%)
	Subtotal	18 (6.7%)
Examination/Assessment	Incorrect diagnosis	8 (3%)
	Investigation undertaken to detriment of patient	5 (1.9%)
	Significant pathology missed	6 (2.2%)
	Case history inadequate, missed secondary condition	4 (1.5%)
	Over-exposure of film	1 (0.4%)
	Failure to request X-ray report	1 (0.4%)
	Failure in referral process	1 (0.4%)
	Other	11 (4.1%)
	Unspecified	2 (0.7%)
	Subtotal	39 (14.6%)
Treatment/Management	Patient experienced post-treatment distress/pain	76 (28.4%)
	Wrong positioning of patient during treatment	5 (1.9%)
	Patient experienced significant post-treatment effects e.g. neurological problem, disc prolapsed	14 (5.2%)
	Patient experienced negative effects during treatment e.g. fracture rib or clavicle	22 (8.2%)
	Suggested drugs to patient which had adverse effect	1 (0.4%)
	Did not modify treatment plan to take account of patient preferences or health needs	8 (3%)
	Slow to refer after patient did not respond to treatment	2 (0.7%)
	Other	13 (4.9%)
	Unspecified	6 (2.2%)
	Subtotal	147 (54.9%)
Other	Unspecified	14 (5.2%)
	Subtotal	14 (5.2%)
Total SIs		268

**Fig. 2:**
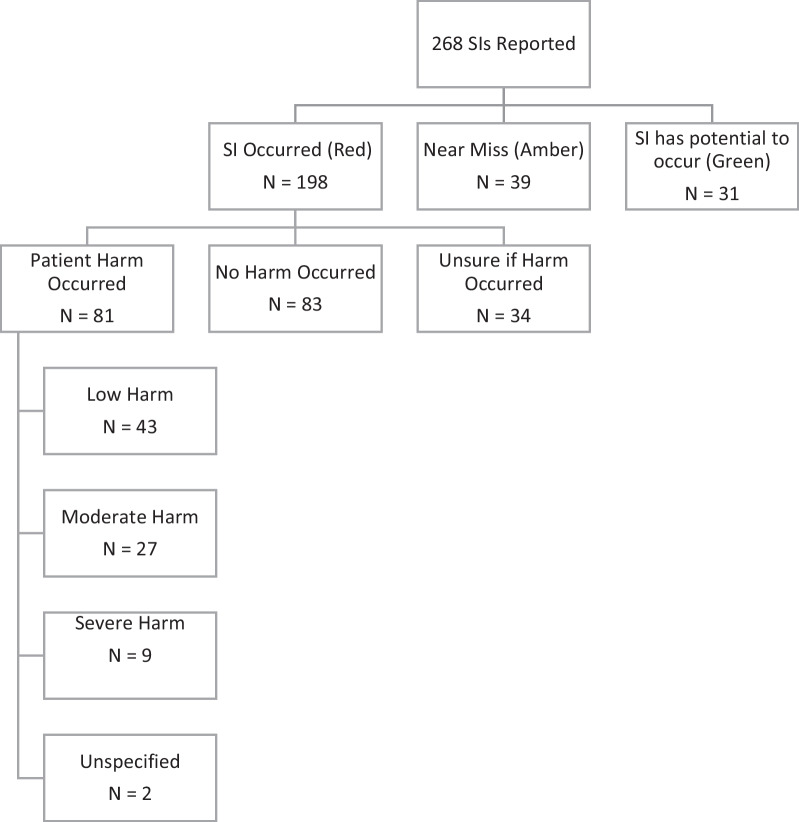
Safety incident type and harm

The distribution of SI types and harm reported to have occurred is shown in Fig. 2. Of the 81 (30.2%) SIs in which patient harm was reported to have occurred (red incidents), 42 SIs were perceived by the reporting chiropractor to be avoidable, and in 51 SIs the reporting chiropractor perceived their actions or inactions were likely to be responsible for the patient harm that occurred.

### Identification of key areas for patient safety improvement

Seven areas for patient safety improvement were identified.

#### Patient trip/fall subcategory

Twenty-three (8.6%) SIs were subcategorised as a patient trip or fall. An additional six incorrectly subcategorised SIs relating to a trip or fall were identified through searching the database with the key words search. Based on the researcher’s analysis of the related SIs, the majority of SIs involved the patient (potentially) falling off the chiropractic bench whilst rolling or transferring from the bench. A common situation involved the chiropractor not supervising the patient during this time. Where SIs reported patients ages, approximately half involved patients over the age of 65. Seven trips and falls actually occurred (Red SIs) and resulted in patient harm. In all seven of these SIs, the chiropractor reported that the incident was avoidable.

#### Post-treatment distress/pain subcategory

The largest single subcategory overall related to post-treatment distress or pain, which accounted for over one quarter of all SIs. Of these, 21 were associated with the cervical spine, of which 11 described neurological symptoms e.g., dizziness, following some form of treatment. Spinal manipulation was reportedly used in the majority of cases (n = 14); however, it is unclear whether the patient also received additional treatment modalities. Twenty-one SIs were reported following treatment of the lumbopelvic region; pain was the most common reaction described. Based on the researcher’s analysis of the related SIs, almost half of these cases (n = 10) described soft tissue techniques as the modality most likely to be associated with the post-treatment experience. This was most commonly described as ‘trigger point therapy to the gluteal region’ resulting in a combination of pain as well as localised bruising. The majority of patients experiencing pain following soft tissue treatment were female (n = 8) and all patients were over 45 years old. A further nine SIs reported post-treatment distress or pain following the use of acupuncture or dry needling.

#### Negative effects during treatment subcategory

Twenty-two (8.2%) incidents were recorded under the subcategory of ‘patient experienced negative effects during treatment e.g. fractured rib or clavicle’. Following a key word search, a total of 26 SIs involving some type of rib injury associated with treatment were identified. Fourteen of these indicated that a rib fracture was likely to have occurred. Available patient and treatment characteristics are summarised in Table 3.

**Table 3:** Incidents involving a suspected rib fracture

Patients gender	Patients age	Consideration of patients bone mineral density (BMD)	Treatment technique	Onset following treatment
Female	55–64	No indications of osteoporosis identified	Lumbar manipulation - side posture	Immediate
Female	45–54	Patient stated “borderline osteoporosis”	Thoracic manipulation - prone	Immediate
Female	75 +	Undisclosed	Thoracic manipulation - prone	Within 1 h
Male	75 +	Undisclosed	Thoracic manipulation - prone	Immediate
Male	65–74	Undisclosed	Thoracic manipulation	Undisclosed
Male	55–64	Undisclosed	Thoracic manipulation - supine	Immediate
Female	65–74	Undisclosed	Thoracic manipulation	Immediate
Female	45–54	Patient stated she did not have osteoporosis	Thoracic manipulation - prone	Immediate
Female	55–64	Undisclosed	Thoracic manipulation - prone	Immediate
Female	45–54	Undisclosed	Thoracic manipulation - prone	Immediate
Female	55–64	Patient on long term steroid mediation	Thoracic manipulation - with drop	Within 24 h
Female	45–54	Osteopenia confirmed (following referral)	Thoracic manipulation - prone	Immediate
Female	55–64	Undisclosed	Sacral drop - prone	Undisclosed
Female	55–64	Undisclosed	Lumbar manipulation - side posture	Undisclosed

Analysis of the related SIs on rib injuries reveal some apparent trends. Most of the suspected rib fractures occurred in female patients. All patients affected were over the age of 45 years with the modal age group being 55–64 years. Most suspected rib fractures involved manipulation directly to the thoracic spine (n = 11). Eight SIs stated the patient positioning during thoracic spine manipulation, with seven of these describing the patient in the prone position. Two SIs involved a lumbar side posture manipulation resulting in a suspected rib fracture. The majority of SIs involving a suspected rib fracture did not disclose a consideration of the patient’s risk of osteoporosis.

#### Significant post-treatment effects subcategory

In total, 18 SIs reported significant post-treatment effects e.g. neurological symptoms, although two of these were associated with assessment rather than treatment. Several clinicians described actions taken following the incident to learn from the event and reduce the risk of recurrence. However, 11 did not document any learning or action taken from the SI. Chiropractors often perceived a common contributing factor was inadequate history taking (in relation to relevant medical/family history).

#### Syncope (new theme)

A theme relating to patients fainting or feeling faint was developed. Sixteen (5.9%) SIs related to this theme were reported. Half of these incidents (8) occurred before treatment, commonly at the examination phase, with two of these occurring during a blood pressure reading. Half of the incidents (8) occurred during treatment, two of which did not state the treatment modality used and one described cervical SMT. The other 5 described acupuncture/dry needling. Nine involved female patients (one of which was pregnant) and the remaining seven involved male patients. Actions taken were variable but normally involved monitoring the patient after the incident. The main learning points suggested by the reporting clinicians were not leaving a patient unsupervised as well as lying the patient down immediately.

#### Failure to recognize serious underlying pathologies (new theme)

A theme of (potentially) missing a serious underlying pathology was developed, representing a total of 22 (8,2%) SIs. Most chiropractors demonstrated some form of personal reflection when recording the SI. However, seven reported SIs did not include any evidence of learning or action taken to mitigate further risk of (potential) patient harm. A commonly recorded perceived contributing factor was absent, delayed or inappropriate referral to another healthcare professional or directly for imaging. Other factors included inadequate history taking or examination as well as a positive response to care masking underlying pathology.

#### Continuity of care (new theme)

Another theme developed was a break in continuity of care i.e. a patient’s care involving more than one practitioner. A total of 14 (5.2%) SIs reported such incidents. These SIs mostly involved some form of locum cover (n = 12); either a different chiropractor within the clinic or an external locum. Most commonly (n = 5) the patient experienced some form of post-treatment distress/pain. It was perceived that a lack of familiarity with the patient’s case was a contributing factor e.g. delivering care in a different way. On reflection, chiropractors felt that there were signs the patient was uncomfortable with having care from another chiropractor, which should have triggered ceasing or modifying care.

## Discussion

This analysis of 10 years of safety incident reporting through CPiRLS, identified 268 SIs, with learning documented in 143 (53.4%) of these. Characterisation of the nature of incidents reported revealed that the majority were ‘red’, indicating that the SI had occurred. The harm levels reported were variable, with nine red SIs being reported as ‘severe’ harm, 42 SIs being reported as ‘avoidable’ and 51 reported as being caused by the chiropractor’s action/inaction, suggesting targets for safety improvements. The final objective of the study was to identify key areas for safety improvement, and seven areas were identified. The risk of trips/falls in a chiropractic clinic and suspected rib fractures following manual therapy are established risks with patient safety notices published in 2009 [21]. The thematic analyses also enabled the development of new areas for safety improvement, demonstrating the value of this approach. The new knowledge from this study should now be disseminated to the profession, by issuing new and updated safer practice notices related to the key areas identified. A further advantage of the new knowledge gained is to enable refinement to CPiRLs to more efficiently capture data regarding these SIs in the future.

Fluctuations in yearly reporting rates on CPiRLS were noted although, on average the reporting rate increased, suggesting more chiropractors are engaging with the system over time. Of note, several other reporting systems exist within individual ECU member countries that enable chiropractors to record SIs [22]. This may account for the lack of engagement with CPiRLS in some ECU member countries. Expected reporting rates are difficult to benchmark, however, given the known high incidence of benign post-treatment AEs associated with manual therapy [11], this study suggests significant under-reporting. Under-reporting is a significant limitation of IRSs in general, with a low sensitivity of incident reporting being observed [23]. CPiRLS relies on chiropractors to proactively report patient SIs rather than actively screen for them. A recent RCT comparing two IRSs identifying AEs associated with chiropractic management found a significant difference in the reporting rate of AEs. In that study, CPiRLS was used as a control and produced significantly lower reporting rates (0.1%) compared to active surveillance involving questionnaires completed by the chiropractor and patient following each appointment (8.8%) [19].

Whilst a positive attitude towards patient safety has been observed within the chiropractic profession, SI reporting remains an unlikely course of action [24]. A lack of engagement with SI reporting is common across healthcare [25, 26]. Barriers to SI reporting within the chiropractic profession have previously been reported, including time requirements and lack of clarity on reporting [27, 28]. Chiropractors, like other private practitioners, are reimbursed based predominantly on patient contact time. Clinical governance activities such as SI reporting require time that is not directly reimbursed. This is a difficult barrier to overcome without direct incentives in place to encourage behaviour change. Other barriers to SI reporting may be modifiable, such as clinician understanding of incident reporting. Education may have an important role in addressing misconceptions and uncertainties about SI reporting within the chiropractic profession. Some chiropractors may not be aware that CPiRLS is completely anonymous with appropriate online encryption in place.

In this study, we found that most cases documented some aspect of learning, however, the emphasis of SIs in the database was often on reporting rather than learning. Whilst SI reporting is important, learning must also occur to improve patient care for an IRS to be deemed effective [8]. In a recent registrant survey within the UK, the majority of chiropractors (58%) stated their workplace used an IRS, ranging from the CPiRLS to local clinic systems. These registrants stated that regular discussions about SIs are carried out to improve patient safety. Some participants mentioned checking the CPiRLS database directly to learn from SIs to improve patient safety [10]. The lack of documented learning may reflect the CPiRLS current format rather than chiropractors’ attitudes and behaviours towards incident reporting and learning. Setting parameters and expectations around data entry may improve the quality of data available for future analysis and subsequent learning.

### Patient safety improvement

The risk of patient falls is well known within healthcare, with the potential for severe harm. Within the UK National Health Service (NHS), as recorded on the National Reporting and Learning System (NRLS), falls are the most common SI reported [29]. This data is based predominately on hospital settings for which it has been demonstrated that 20–30% of falls can be prevented through appropriate risk management [30]. It is important for chiropractors to be aware of the risk of patient falls and undertake appropriate risk assessments with mitigating measures in place to reduce the risk of occurrence. In particular, it is important to ensure appropriate patient positioning and supervision of the patient during transitions involving the treatment table.

Fainting (syncope) had not been previously identified as a risk and is not currently a reporting subcategory within CPiRLS. Fainting (or feeling faint) has been identified as a common, benign AE associated with cervical manipulation [31] and acupuncture treatment [32], but should also be considered at other points in the clinical encounter. Fainting is very common within the population and may occur due to a variety of reasons [33]. An important consideration would be adequate first aid training to develop the competence and confidence to manage patients who have fainted or feel faint.

Due to the frequency of benign AEs associated with manual therapy, it is not surprising that the largest category on the CPiRLS database was treatment/management. It is difficult to establish a causal relationship between manual therapy and AEs due to a number of confounding variables including: presence of underlying pathology [34], patient treatment beliefs [35], contextual effects and the natural history of musculoskeletal pain [36]. In addition, clinicians and patients appear to have different perceptions of what defines an AE [35, 37]. It is notable that some chiropractors perceived reported AEs as having resulted from treatment, and categorised and described them as such. Other chiropractors reported that an underlying pathology was present, and the natural history of the pathology resulted in a temporal association between treatment and an AE.

The design of CPiRLS does not enable causality to be established in the relationship between manual therapy and AEs, however analysis of the database has identified key points for reflection and learning to reduce the risk of occurrence of AEs. AEs were reported at all stages of patient management, including assessment, and were associated with all forms of manual therapy. In particular, benign AEs in the lumbopelvic region where often reported to be related to soft tissue treatment. A study that reviewed the risk of care in the UK osteopathy profession concluded that particular types of treatment including manipulation were not related to outcomes, including AEs [38]. There may be a limited awareness among practitioners and the public about the risks of non-manipulative techniques associated with AEs. Patients should be adequately informed about the currently established risks associated with manual therapy to ensure informed consent is gained and shared decision making can occur.

In our study, AEs associated with the thoracic spine related to harm to the ribs. Rib fracture following spinal manipulation is a known risk, however, there is limited literature available on this topic. A recent qualitative case series described three incidents of rib fracture following manipulation, only one of the three cases described a prone technique being performed [39]. However, our analysis of CPiRLS data suggests a possible association between prone thoracic spine manipulation and rib fractures. This requires further investigation since, to our knowledge, this has not been previously proposed or evaluated as a risk factor. Our analysis also highlighted that a suspected rib fracture is a potential outcome following a range of manual therapy techniques and is not limited to manipulation of the thoracic spine. This study has added new knowledge to our understanding of rib fractures associated with manipulation of the spine, but more research is required to understand any potential risks involved.

Osteoporosis is regarded as a relative contra-indication to manipulation due to the increased risk of fragility fractures [40], and it is possible that this may have contributed to the fractures reported on CPiRLs. However, most chiropractors did not document any consideration of the patient’s bone mineral density (BMD) in the cases where rib fractures were reported as SIs. Before considering manual therapy, patients should be adequately and periodically screened for osteoporosis and the risk of fragility fractures. Osteoporosis is commonly associated with older age i.e. postmenopausal patients, however a number of the reported rib fracture incidents involved patients in a lower age group i.e. 45–54. The RCC has recently published a Chiropractic Quality Standard on the management of osteoporosis within a chiropractic care setting [41]. This standard recommends all patients over 40 should be periodically assessed for the possibility of osteoporosis. In addition, manual therapy forces should be modified to reduce the risk of harm to patients at risk of osteoporotic fractures. Previous CPiRLS data analysis did not identify the risk of rib fractures for patients in this lower age range. A benefit of periodically analysing CPiRLS data is that the increasing accumulation of data allows safer practice notices and other resources to be developed to enhance patient safety.

In relation to the cervical spine, this study found that benign AEs involving neurological symptoms were experienced by patients in addition to pain. A secondary analysis of a cross-sectional survey of patients receiving manual therapy found treatment to the neck has the greatest number of symptoms perceived as an AE [35]. This finding is theorised to be due to patient treatment beliefs around manual therapy to the neck, influenced by increased awareness of serious complications i.e. frequent media coverage of neurovascular events [35]. Cervical manipulation was described in the majority of cases associated with benign AEs on the CPiRLS database, however, details of reported treatments were limited. Cervical manipulation may present an increased risk for benign AEs, however the literature is not clear [11]. It is advisable for chiropractors to always consider the appropriateness of different treatment modalities in shared decision making with patients. For example, consideration of cervical manipulation in light of the potential association with increased risk but lack of superior efficacy compared to other approaches [42].

A controversial association between cervical manipulation and serious AEs, such as neurovascular events, continues to exist [43]. In the case of neurovascular events, a temporal association may be more likely i.e. a patient with underlying vascular pathology of the neck presenting with neck pain and/or headaches [14, 34]. Two SIs identified in the CPiRLS database that reported potential neurovascular events were associated with assessment and stated that treatment did not occur. This highlights the possibility of patients presenting with underlying vascular pathologies and experiencing a neurovascular event in the absence of cervical manipulation.

Chiropractors reporting SIs often reflected on the importance of case history taking when, in retrospect, the patient was shown to be presenting with serious underlying pathology. To enhance patient safety, patients should always be screened for masqueraders of musculoskeletal pain (including vascular pathologies of the neck) through appropriate case history taking and physical examination [44]. Consideration should also be given to continuity of care during the management of patients. Analysis of the CPiRLS database has highlighted that lack of an established relationship between the practitioner and patients may present a risk for SIs, including missing a serious underlying pathology.

### Study limitations

We found that the accuracy of reporting in terms of correct categorisation of SIs was variable. SIs that had not been assigned a category or subcategory accounted for a about one fifth of total cases. Missing data limited the ability to analyse the CPiRLS database. This was apparent in discrete data fields as well as open text fields. A review of the categorisation and reliability of categorisation of incidents is proposed to improve the quality of future reporting.

As only one researcher independently analysed the data, there is further potential for inaccuracies in the analysis. In addition, extraction of details within the SIs could have been unreliable e.g. patient positioning during treatment. However, this was mitigated by only extracting details that were unambiguous.

The severity of harm described in the CPiRLS system has to be interpreted with caution; currently, CPiRLS provides no definition of low, moderate or severe harm. AEs have been defined in relation to manual therapy based on level of severity [4, 45] and it will be important to adapt such a set of definitions into CPiRLS.

Chiropractors will report an incident, on a voluntary basis if they believe an AE or other type of SI has occurred. This is therefore dependent on direct observation of an SI by a chiropractor or patient disclosure. Patients and clinicians may attribute AEs to chiropractic care using different criteria based on their beliefs about manual therapy. Patients may not report symptoms that they do not feel are associated with chiropractic care. Conversely, some rare symptoms experienced by patients following manual therapy e.g. depression have been strongly perceived by patients to be an AE [35]. Symptoms of this nature may not be perceived as relevant AEs by chiropractors and therefore unlikely to be reported onto CPiRLS.

### Future direction of CPiRLS

Wangler et al., developed nine recommended features for the successful establishment of a chiropractic reporting and learning system [46], and these are partly met by CPiRLS. The aim of CPiRLS should be reviewed to prioritise the type of incident reporting and learning that will drive patient safety within chiropractic. An expert panel (The CPiRLS development group) has been appointed by the RCC to further develop CPiRLS and promote its use within the profession, including working with licensee organisations to support them in promoting CPiRLS in their jurisdictions. To encourage the profession to engage with CPiRLS, and to increase the reporting levels and learning from that data, a number of measures are required. These include publishing clearer guidelines and definitions for reporting on CPiRLS, including definitions of severity of harm and refining classification of SIs.

While two trends in the type of incidents reported have been previously identified and evidence-based guidance produced, these safety notices should now be updated based on the findings of this study and recommendations of current guidelines. New notices should be developed and published to educate the profession on the potential risks identified and recommended mitigation strategies. To increase learning from SIs an appropriate strategy may be a mechanism of timely feedback to the individual reporter by the expert panel [46]. The CPiRLS database should continue to be reviewed on a timely and periodic basis, with the results shared with the profession. CPiRLS must also receive continued support from the professional associations and educational institutions to improve the culture of reporting and to drive awareness among students and clinicians.

## Conclusion

This detailed review of ten years of patient SI reporting by the chiropractic profession demonstrates that, while significant under-reporting is highly suspected, there has been an upward trend in the frequency of SI reporting to CPiRLS during the period 2009 to 2019, providing a sizeable database for useful analysis. Some aspect of learning was documented by reporting chiropractors in over half of the SIs. The largest subcategory involved patients experiencing post-treatment distress/pain. Patient harm was reported in 30% of SIs, however, the level of harm is unclear.

The nature of the SIs reported in CPiRLS was characterised. This resulted in the development of seven key areas for patient safety improvement representing a potential to reduce risk to patient safety during chiropractic management. This information needs to be disseminated to clinicians to optimise learning from SIs and facilitate appropriate risk management strategies. To continue to drive the culture of incident reporting and learning forward, several important measures have been identified that need to be actioned by the profession. This evaluation demonstrates that CPiRLS has an important role in improving patient safety in the chiropractic profession.

## Supplementary Information

**Additional file 1**. Reporting a red Incident on CPiRLS.

**Additional file 2**. Additional descriptive statistics of the CPiRLS Database 2009 to 2019.

## Data Availability

Due to ethical concerns, supporting data cannot be made openly available. Further information about the data and conditions for access are available through the Royal College of Chiropractors.

## Abbreviations

AE: Adverse event
BMD: Bone mineral density
CPiRLS: Chiropractic patient incident reporting and learning system
ECU: European Chiropractors Union
IRS: Incident reporting system
NRLS: National reporting and learning system
NHS: National Health Service
RCC: Royal College of Chiropractors
SI: Safety incident
UK: United Kingdom
