# Supplementary material for: Ten years of online incident reporting and learning using CPiRLS: implications for improved patient safety
Source: Chiropr Man Therap. 2023 Feb 15;31:9. doi: 10.1186/s12998-023-00477-1 (PMC9933370; doi:10.1186/s12998-023-00477-1)
Supplement: Supplementary file 1 — Additional file 1. Reporting a red Incident on CPiRLS. [file 12998_2023_477_MOESM1_ESM.docx]

**Additional File 1. Reporting a red Incident on CPiRLS**

**Please provide the patient's age range:**

- Under 16
- 16 – 24
- 25 – 34
- 35-44
- 45 – 54
- 55 – 64
- 65 – 74
- 75+
- Undisclosed

**Please provide the patient's gender:**

- Female
- Male
- Undisclosed

**Where the incident happened:**

- Unspecified
- Patients Residence
- Treatment Room
- X-Ray Room
- Waiting Room
- Rehabilitation Room

**Please provide a category for the nature of the incident:**

- Documentation
- Examination/ Assessment
- Treatment/ management
- Accidents/ Equipment/ Infrastructure
- Other

**Please provide a subcategory:**

**DOCUMENTATION**

- Patient record inadequate
- Failure to take notes on a new episode
- Failure to document diagnosis / prognosis
- Patient record misplaced
- Records confused, treated wrong patient
- Treated before referral notes arrived, missed significant finding
- Failure to gain consent
- Breach of confidentiality

**EXAMINATION/ ASSESSMENT**

- Incorrect diagnosis
- Investigation undertaken to detriment of patient
- Significant pathology missed
- Case history inadequate, missed secondary condition
- Over-exposure of film
- Over-exposure of patient
- Failure to request x-ray report
- X-ray misinterpretation
- Exposure of pregnant patient
- Failure in referral process

**TREATMENT/ MANAGEMENT**

- Patient experienced post-treatment distress/pain
- Wrong positioning of patient during treatment
- Patient experienced significant post treatment effects e.g. neurological problem, disc prolapsed
- Patient experienced negative effects during treatment e.g. fractured rib or clavicle
- Suggested drugs to patient which had adverse effect
- Did not modify treatment plan to take account of patient preferences or health needs
- Slow to refer after patient did not respond to treatment
- Did not discontinue treatment when appropriate to do so
- Patient discharged without arranging future care

**ACCIDENTS/ EQUIPMENT/ INFRASTRUCTURE**

- Patient trip/fall
- Patient unable to contact clinic in an emergency
- Equipment malfunction
- Failure to use equipment appropriately
- Health and Safety measures inadequate
- Failure to dispose of sharps and clinical waste appropriately
- Medical emergency inadequately handled
- Exposure to blood
- Exposure to harmful substances

**What happened - give details, including people and/or equipment involved:**

**Why and how it happened - describe the sequence of events and possible causes:**

**Describe the actions taken immediately and in the longer term:**

**Please provide up to 5 key words to describe this incident:**

**Example 1: thoracic, manipulation, rib, fracture, neoplasm**

**Example 2: LBP increased following massage**

**Was the patient harmed?**

- Unspecified
- Yes
- No
- Don’t Know

If yes - **Please grade the harm:**

- Unspecified
- Low
- Moderate
- Severe

**Could the incident have been avoided?**

- Unspecified
- Yes
- No
- Don’t Know

If yes - **Incident could have been avoided - please give your opinion/ideas:**

**How often have you encountered this type of incident in your practice?**

- Unspecified
- Never
- Once before
- Twice before
- More than twice before

**What is the likelihood that your actions/inactions were responsible for the incident?**

- Unspecified
- Likely
- Unlikely
- Uncertain

**Please provide any further information about the incident that you consider relevant (e.g. medication, concurrent treatment, family history and/or features in the patient's current or past health):**
