# Supplementary material for: Ten years of online incident reporting and learning using CPiRLS: implications for improved patient safety
Source: Chiropr Man Therap. 2023 Feb 15;31:9. doi: 10.1186/s12998-023-00477-1 (PMC9933370; doi:10.1186/s12998-023-00477-1)
Supplement: Supplementary file 2 — Additional file 2. Additional descriptive statistics of the CPiRLS Database 2009 to 2019. [file 12998_2023_477_MOESM2_ESM.docx]

**Additional File 2. Additional descriptive statistics of the CPiRLS Database 2009 to 2019.**

**Table 1. Number of SIs reported per year**

| **Year** | **Number of SIs** |
| --- | --- |
| 2009 (April – Dec) | 24 |
| 2010 | 9 |
| 2011 | 21 |
| 2012 | 26 |
| 2013 | 23 |
| 2014 | 29 |
| 2015 | 36 |
| 2016 | 32 |
| 2017 | 19 |
| 2018 | 38 |
| 2019 (Jan – March) | 11 |
| **Total** | **268** |

**Table 2. Number of SIs reported for each patient age group**

| **Patient's Age** | **Number of SIs** |
| --- | --- |
| Under 16 | 6 (2.2%) |
| 16 – 24 | 6 (2.2%) |
| 25 – 34 | 27 (10.1%) |
| 35-44 | 41 (15.3%) |
| 45 – 54 | 43 (16.0%) |
| 55 – 64 | 54 (20.1%) |
| 65 – 74 | 39 (14.6%) |
| 75+ | 19 (7.1%) |
| Undisclosed | 33 (12.3%) |
| **Total** | **268** |
